# Plastic Cutlery Alternative: Case Study with Biodegradable Spoons

**DOI:** 10.3390/foods10071612

**Published:** 2021-07-12

**Authors:** Dani Dordevic, Lucie Necasova, Bojan Antonic, Simona Jancikova, Bohuslava Tremlová

**Affiliations:** Department of Plant Origin Food Sciences, Faculty of Veterinary Hygiene and Ecology, University of Veterinary Sciences Brno, Palackeho tr. 1946/1, 612 42 Brno, Czech Republic; h18175@vfu.cz (L.N.); antonicb@vfu.cz (B.A.); jancikovas@vfu.cz (S.J.); tremlovab@vfu.cz (B.T.)

**Keywords:** plastics, biodegradable spoons, texture, antioxidant activity, polyphenols

## Abstract

Plastics are mixtures of organic polymers that play a major role in environmental contamination worldwide. One way to reduce the waste arising from the use of plastics, especially disposable ones, can be to produce environmentally friendly cutlery. The aim of the work was the production of biodegradable spoons and evaluation of their texture, antioxidant activities and total polyphenols content. The spoons were made from a combination of the following ingredients: water, grape, proso millet, wheat, xanthan and palm oil in different concentrations. The samples were baked at 180 or 240 °C, some spoons were dried in a fruit dehydrator. According to the results of the analysis, a spoon prepared from a mixture of all three flours and with the addition of xanthan appears to be the most suitable replacement for plastic cutlery. This spoon showed high strength and antioxidant activity. It was confirmed that the use of grape flour has a beneficial effect on the nutritional profile of the experimentally produced biodegradable spoons.

## 1. Introduction

At the present time, plastics are a much-discussed topic, as their use and production are constantly increasing and accumulating in an environment where they persist for hundreds of years and become dangerous not only for the environment, but also for humans. Due to external influences, plastic products break down into microplastics and enter the sea, where they endanger aquatic animals [1,2]. Disposable plastics have become the most widely used material in the daily life of most households. They gained popularity due to their versatility, low price and weight. Thermoplastics polypropylene and low density polyethylene are mostly used for their production [3].

The European Commission has responded to the increased consumption of disposable plastics by announcing Directive (EU) 2019/904 on the reduction of the environmental impact of certain plastic products [4]. The Member States of the European Union are thus gradually incorporating this directive into their legislation. The sale of products for which there is an alternative should be prohibited. This applies to plastic cutlery, plates, straws, cotton swabs, drink mixers, food packaging made of polystyrene and oxo-degradable plastic products. For other disposable plastic products, measures should be taken to reduce their consumption. At the same time, they must be supplemented with information on disposal methods and negative effects on the environment. The Directive also sets out requirements for PET bottles made of polyethylene terephthalate. Within the Union, this is the most frequently found waste on beaches. By 2025, these bottles should be at least 25% recyclable. The ban is to apply in the Czech Republic from July 2021 [4].

With the growing use of plastics, especially disposable ones, and with greater consumer awareness of their negative impact on the environment, various alternative products have begun to be produced. Many entrepreneurs today are trying to replace disposable plastics with reusable products or with more eco-friendly products [5].

The aim of the study was to find a suitable recipe for biodegradable spoons, which would be an adequate substitute for plastic cutlery. Emphasis was placed on finding a recipe with favorable textural and nutritional parameters.

## 2. Materials and Methods

**Figure 1:**
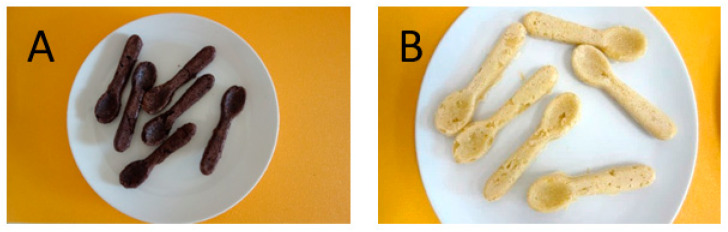
The examples of biodegradable spoons with (**A**) and without (**B**) grape seed flour.

Biodegradable spoons were prepared from three types of flour in various combinations and concentrations. The following flours were used: wheat flour (Mlýny J. Voženílek, spol. s r.o., Předměřice nad Labem, Czech Republic), millet smooth flour (brand: Natural) and grape flour (brand: Health from Nature). All ingredients were mixed to form a firm dough which was filled into a silicone spoon mold. The spoons were baked for 10 min at 180 °C (oven ELENA, UNOX, Cadoneghe, Italy). Four batches of experimentally produced biodegradable spoons were baked at 240 °C. A total of 32 batches of biodegradable spoons were prepared. The full information about experimentally produced biodegradable spoons is shown in Table 1. The experimentally produced spoons were dried at 60 °C during 60 min (used drier: SNACKMAKER FD500, Liberec, Czech Republic). The examples of experimentally produced biodegradable spoons are shown in Figure 1.

The texture (hardness) of experimentally produced biodegradable spoons was measured before and after drying. According to the textural properties, six batches of experimentally produced biodegradable spoons with the highest hardness were evaluated by the following chemical analyses: total phenol content (TPC), ferric reducing antioxidant power (FRAP) and 2,2-diphenyl-1-picrylhydrazyl (DPPH). The hardness of biodegradable spoons was compared with four different plastic spoons purchased in Brno (Czech Republic > Tesco shop): the labeling of plastic spoons indicated that they were composed of polystyrene and that they were intended for use in the temperature range from −10 to + 70 °C. The colors of plastic spoons were white and green (big green spoons: Table 2).

### 2.1. Textural Parameters

The textural properties were measured by texturometer TA.XTplus Texture Analyser (Goldaming, Surrey, UK, software: Stable Micro Systems, Surrey, UK). A large three-point bending stand with a base measuring 240 × 90 mm was used. Firstly, the instrument was calibrated and then each sample was measured using the following program: “Measurement of the hardness and resistance of biscuits/cookies to bend or snap”; the method in the Exponent software, the software supported by texturometer TA.XTplus (Stable Micro Systems Ltd., Vienna Court, Lammas Road, Godalming, Surrey, UK). All measurements were performed at least five times.

### 2.2. Determination of Antioxidant Activity by DPPH Method

In the DPPH method, ultrasonic extraction of 0.1 g of the homogenized sample in 20 mL of ethanol was performed. Furthermore, 3 mL of ethanol as a blank and, in parallel, 3 mL of the filtered (the filtration done by filter paper Whatman 1) extract were incubated with 1 mL of 0.1 mM DPPH ethanolic solution in the dark for 30 min. After incubation, the absorbance of both solutions was measured on a spectrophotometer at 517 nm, and the results were expressed as the percentage of inhibition (degree of color loss) for free radical DPPH with a sample against the blank [6]. All measurements were performed in triplicate.

### 2.3. Determination of Antioxidant Activity by FRAP Method

The FRAP method was implemented by the ultrasonic extraction of 0.1 g of the homogenized sample in 20 mL of 75% methanol, after which the 180 µL filtered extract (the filtration done by filter paper Whatman 1) with the addition of 300 µL of distilled water was incubated with 3.6 mL of working solution (acetate buffer + TPTZ + FeCl_3_ × 6H_2_O in ratio 10:1:1) for 8 min in the dark and then measured for absorbance at 593 nm on a spectrophotometer (CE7210, Cambridge, UK). The obtained results are expressed in µmol Trolox/g since Trolox was used for making the calibration curve [7]. All measurements were performed in triplicate.

### 2.4. The Total Polyphenols Content

The total polyphenols were obtained after extraction of 1 g of the sample in water during 10 min of shaking. Then, 1 mL of the filtered extract was mixed with 5 mL of 1:10 Folin–Ciocalteu/water solution as well as 4 mL Na_2_CO_3_ (75 g/L), and incubated in a 25-mL volumetric flask in the dark for 30 min. After incubation, the flask was filled to the designated mark and the optical density of the solution was measured at 765 nm. The results are expressed as mg/g of gallic acid equivalent (GAE) for the reason that gallic acid was used for obtaining the calibration curve [8]. All measurements were performed in triplicate.

### 2.5. Statistics

The results of the analyses were statistically evaluated using the IBM SPSS Statistics 12 computer program. Statistical significance at the level of *p* < 0.05 was evaluated by one-way ANOVA, the Tukey post-hoc parametric test (when Levene’s test showed equal variance, *p* > 0.05) and the Games–Howell post hoc nonparametric test (when Levene’s test showed unequal variance, *p* < 0.05) were used to find differences within groups.

## 3. Results and Discussion

### 3.1. Textural Parameters

The texture parameters of plastic spoons are shown in Table 2. Four types of plastic spoons were used for the measurement of hardness. According to the European directive 904/2019 [4], from July 2021, plastic spoons will be restricted and will not be sold in the European countries.

The comparison of experimentally produced biodegradable spoons with different xantan concentrations is shown in Table 3. Thanks to its polymer structure and hydrophilic properties, xantan serves as a perfect binder. The resulting solutions are resistant to high temperatures, and therefore xanthan gum is suitable for the preparation of baked products [9,10]. Though, from the Table 3 it can be perceived that hardness increased statistically significantly (*p* < 0.05) with a higher amount of xantan only for the sample 2PX1 ^S^ (the dried sample with lower content of millet flour).

The analysis showed that a sample with the same composition, but not dried, achieved a higher hardness with the addition of 2.5 g of xanthan (1522.01 ± 320.51 g) compared to a formulation with 5 g (1220.30 ± 505.66 g). A statistically significant (*p* < 0.05) difference was found between 2PX1 ^S^ and 3PX1 ^S^ samples (Table 3). In 2018, Encina-Zelada et al. [11] studied the effect of xanthan gum on the physico-chemical, rheological, and textural properties of gluten-free bread. The study showed that a greater addition of xanthan a mixture of rice, corn and quin flour resulted in lower stickiness and cohesiveness of the dough, but the dough was still firmer, with good consistency and viscosity. The best results were achieved by loaves with an amount of xanthan of 1.5–2.5% and with 110% addition of water [11]. Certainly, the texture parameters of experimentally produced biodegradable spoons were affected by the presence of so-called “gluten” proteins—prolamins and glutelins, able to create a harder texture [12].

Drying at high temperature (60 °C) had a great influence on the hardness of the spoons. During drying, water is removed, and the product becomes more durable and stronger [13]. Samples (2PX1 ^x^ and 3PX1 ^x^) containing xanthan were baked at two different temperatures, at 180 and 240 °C for 10 min.

The sample with a lower addition of xanthan (2.5 g) showed a lower hardness (1522.01 ± 320.51 g) than a sample containing 5 g of xanthan (1971.62 ± 908.61 g). The highest hardness was reached in the 3PX1 sample (2763.61 ± 345.08 g), baked at 240 °C. The results suggest that the hardness increased with a higher temperature. Likewise, a significant effect of xanthan on textural properties was demonstrated too. A statistically significant difference was found between a sample with 2.5 g of xanthan, baked at 180 °C, and a sample with 5 g of xanthan, baked at 240 °C. The dough changes its structure irreversibly during baking. The first changes occur at a temperature of 15–40 °C, when the fat melts. At 70 °C, the starch begins to gelatinize, and as the temperature rises to 90 °C, the baked products become firmer. Water evaporates when it exceeds 100 °C and the product loses its original semi-solid consistency [14].

The hardness of biodegradable spoons prepared from gluten free millet flour with different water contents is shown in Table 4.

From the results it can be read that the highest hardness is achieved in samples with 40 mL of water. The highest hardness was shown by the dried sample with the addition of 40 mL of water (1553.75 ± 641.37 g), the lowest hardness was measured in the non-dried sample with 50 mL of water (542.75 ± 68.63 g). Spoons prepared with 20 mL of water were not measured as they disintegrated after baking. There was a statistically significant (*p* < 0.05) difference between dried and non-dried sample (with the addition of 50 mL and 30 mL of water), containing 50 mL of water. Similar results were obtained for samples of spoons with 30 mL of water (Table 4). During baking, the water begins to evaporate at temperatures over 100 °C. In this phenomenon, water gets from the dough to the surface and causes a color change. The color changing affects sensory properties of the product and consequently consumers’ acceptance [14]. According to the study conducted in 2013 by De la Hera et al. [15], the amount of hydration and the grain size of the used flour play an important role in the quality of gluten-free bread (the study used rice flour for analysis). As a result, the product achieved high hardness and low volume at lower hydration.

The texture was also evaluated for samples prepared with and without addition of palm oil. The results are shown in Table 5.

The highest hardness was obtained with the dried sample 1PS (6204.51 ± 2711.98 g), which did not include palm oil in its recipe. In contrast, the lowest hardness was measured for a 3P sample with 10 g of oil. The results showed that the addition of palm oil negatively affected the hardness of experimentally produced spoons (Table 6). Statistically significant (*p* < 0.05) differences between samples with and without oil addition were found between the following samples: 2P, 1PS and 2PS.

Samples containing xanthan were analyzed for the effect of palm oil addition on spoon hardness. The results are shown in Table 7. A statistically significant difference (*p* < 0.05) was observed for the 2PX1 ^yS^ sample, the other results were not statistically significant (*p* > 0.05).

Higher values were obtained for dried samples, not including palm oil in their recipe. The addition of 5 g of oil resulted in a relative reduction in hardness. Conversely, this was the case for non-dried samples. The highest hardness of the 2PX1 ^yS^ sample was mainly due to drying at 60 °C, but the addition of 5 g of xanthan was also significant, helping to create stable products with optimal structure. The results showed that the presence of more xanthan increases the hardness of the spoons only after drying. Fat is added to the dough to improve the texture and taste of the product. After baking, the spoons had a more attractive appearance [14].

In their study examining the effect of dough mixing time on the properties of biscuits, Manohar and Rao [16] state that as the fat level increases, the strength of the biscuits decreases and, conversely, their brittleness increases. This statement is in the accordance of studies finding that triacylglycerols significantly affect the crystallization processes, and consequently increases hardness [17].

The hardness of biodegradable spoons with different ratios of smooth wheat and grape flour is shown in Table 7.

The same ratio (1:1) of smooth wheat flour and grape flour showed the highest hardness. From the results it can be read that higher addition of grape seed flour resulted in a lower hardness of the spoons.

Samples of biodegradable spoons with the highest hardness were selected and subsequently their recipe was improved by the addition of grape flour in various concentrations (0%, 5%, 10% and 20%), the obtained results are shown in Table 8 (the samples were baked at 240 °C for 10 min). The highest hardness was reached in spoons with 5% addition of grape flour, that were dried. An exception is the 9^S^ sample, that reached the highest hardness with 10% addition of different amounts of grape flour were found within samples 9, 11S and 12 (Table 8). Grape flour was added to the recipe due to the significant amount of bioactive compounds, especially fiber and polyphenols, substances with antioxidant and anti-inflammatory effects [18]. Ross et al. [19] carried out an analysis examining the acceptability of bread that was enriched with different concentrations of grape flour (10%, 7.5% and 5%). The lowest grape seed flour concentration 5%, proved to be the most suitable amount. A higher proportion of grape flour. The reason is the lower proportion of millet flour in the recipe compared to other examined samples. Statistically significant differences (*p* < 0.05) between the samples has a negative effect on sweetness and causes a feeling of bitterness.

### 3.2. Antioxidant Profile of Biodegradable Spoons

The amounts of total polyphenols and antioxidant activity in selected samples of spoons that reached the highest hardness are shown in Table 9.

Statistically significant differences (*p* < 0.05) were found in almost all detected values of polyphenols, only the samples 2PX1 ^y^ and 9 ^y^ did not differ significantly. The highest proportion of polyphenolic substances was achieved in the sample 11 ^z^ (0.038 ± 0.00 mg/g), prepared from a higher amount of millet flour, with 20% addition of grape seed flour and 5 g of xanthan. The baking temperature was 240 °C. Conversely, the lowest amount of polyphenols was found in the sample 1P (0.007 ± 0.00 mg/g), prepared only from plain and millet flour in the same ratio, baked at 180 °C. The results show that the enrichment of the products with grape seed flour significantly (*p* < 0.05) affected the amount of polyphenols in the final product. This finding is in accordance with the study examining the effect of adding grape seed flour to waffles [20]. The increased amount of polyphenols in sample 11 ^z^ is also due to the addition of millet flour, made by grinding millet. The study showed that millet crop is rich in phenolic compounds, including antioxidants and beta-glucans [21].

The highest DPPH value was found in sample 11 ^z^ (25.91 ± 0.10%), on the contrary, samples 1P and 2PX1 ^y^ showed zero values. The FRAP method showed the highest antioxidant activity in the sample 11 ^z^ (21.57 ± 0.14 µmol/g), the lowest value was shown in the sample 2PX1 ^y^ (0.73 ± 0.04 µmol/g). Statistically significant (*p* < 0.05) differences were found between all tested samples.

From the obtained data it can be concluded that grape flour had a significant effect on the antioxidant activity and the amount of polyphenols. This fact is in accordance with an experiment from 2021, examining the effect of the addition of grape flour in concentrations of 1%, 3%, 5% and 10% on the antioxidant activity and total polyphenols [20]. The most acceptable samples were with 10% addition of flour. This concentration did not fundamentally affect the sensory properties of the product [20]. The study conducted by Levent et al. [22] found that the grape seeds had higher antioxidant properties compared to rice flour, chickpea flour, carrot flour, flaxseeds, pomegranate seeds and poppy seeds. Nakov et al. [23] found that the addition of 4%, 6%, 8% and 10% of grape pomace powder statistically (*p* < 0.05) increased the antioxidant activity of cakes.

The previous study showed that when heated to temperatures higher than 180 °C, the product loses a significant amount of total polyphenols and also reduces its antioxidant properties [19]. The amount of catechin and epicatechin decreased with increasing temperature. On the contrary, there was an increase in gallocatechin and gallic acid. It was also evidenced by the study that antioxidant activity is related to contents of catechin and epicatechin [19].

## 4. Conclusions

The study showed certain indications in biodegradable spoons production recipe and technological production that can serve as starting points for future work. The hardness was affected also by roasting temperature, since hardness was higher in samples roasted at 240 °C in comparison with experimentally produced spoons roasted at 180 °C. The addition of grape seed flour resulted in increased antioxidant properties of spoons. Xantan gum increased the hardness of spoons. The best results were achieved by a sample prepared from smooth, millet and grape flour, which was added in a concentration of 20%, and xantan gum (samples abbreviations: 2PX1 ^S^, 3PX1, 1P ^S^, 2PX1 ^yS^, 2PX1 ^xS^, 9 ^S^, 11 ^S^, 12 ^S^). This highlighted the possibility of grape seed flour to supply the products with the necessary antioxidants and also the importance of xanthan to create the desired texture. Certainly, it is important in future research to determine the sensory acceptability of biodegradable spoons. The importance of the study can be overviewed by the fact that biodegradable cutlery represents an ecological way toward plastic reduction in daily life. Certainly, biodegradable spoons produced in this experiment in this form would not be probably adequate for all food commodities and they would not be reusable, since they could be consumed, but further studies will aim to improve this property too.

## Figures and Tables

**Table 1:** Information about biodegradable spoon ingredients.

Sample	Flour (g)			Water Content (g)	Oil Content (g)	Xanthan Content (g)	Temperature (°C)
	Grape Seed	Wheat	Millet				
1 ^x^	50	50	-	100	-	-	180
1 ^y^	17	50	-	50	-	-	180
1 ^z^	50	17	-	50	-	-	180
1P	-	33	33	50	-	-	180
2P	-	50	17	50	-	-	180
3P	-	17	50	50	-	-	180
1P ^p^	-	33	33	50	10	-	180
2P ^p^	-	50	17	50	10	-	180
3P ^p^	-	17	50	50	10	-	180
4	-	-	66	50	-	-	180
4 ^x^	-	-	66	40	-	-	180
4 ^y^	-	-	66	30	-	-	180
4 ^z^	-		66	20	-	-	180
4 ^p^	-		66	50	10		180
2PX1 ^x^	-	50	17	50	-	2.5	180
2PX1 ^y^	-	50	17	50	-	5	180
3PX1 ^x^	-	17	50	50	-	2.5	180
3PX1 ^y^	-	17	50	50	-	5	180
2PX1 ^xp^	-	50	17	50	5	2.5	180
2PX1 ^yp^	-	50	17	50	5	5	180
9	-	50	17	50	-	2.5	240
9 ^x^	3.4	50	17	50	-	2.5	240
9 ^y^	6.7	50	17	50	-	2.5	240
9 ^z^	13.4	50	17	50	-	2.5	240
11	-	17	50	50	-	5	240
11 ^x^	3.4	17	50	50	-	5	240
11 ^y^	6.7	17	50	50	-	5	240
11 ^z^	13.4	17	50	50	-	5	240
12	-	17	50	50	20	2.5	240
12 ^x^	3.4	17	50	50	20	2.5	240
12 ^y^	6.7	17	50	50	20	2.5	240
12 ^z^	13.4	17	50	50	20	2.5	240

Small letters (^x^, ^y^, ^z^, ^p^) in the superscript indicate the samples batch showed in other Tables.

**Table 2:** Textural characteristics of plastic spoons.

Plastic Spoons	Hardness (g)
Transparent small spoon	4383.34 ± 82.3 ^a^
Green big spoon	9941.46 ± 2419.05 ^b^
White big spoon	4358.73 ± 400.41 ^a^
White small spoon	4066.27 ± 228.70 ^a^

Different lowercase letters (^a^, ^b^) are indicating statistically significant (*p* < 0.05) differences.

**Table 3:** Textural results of biodegradable spoons prepared with different amounts of xanthan.

Sample	Hardness (g)	
	2.5 g Xanthan ^x^	5 g Xanthan ^y^
2PX1	1522.01 ± 320.51 ^A^	1220.30 ± 505.66 ^A^
2PX1 ^S^	5951.24 ± 2395.60 ^aB^	8003.48 ± 5160.20 ^bC^
3PX1	1971.62 ± 908.61 ^aC^	1470.25 ± 377.66 ^bA^
3PX1 ^S^	5204.77 ± 1580.41 ^D^	5117.08 ± 1097.69 ^B^

Letter ^S^ in the superscript indicates the dried samples. Small letters (^x^, ^y^) in the superscript indicate the sample labeling according to the Table 1. Lowercase letters (^a^, ^b^) in the superscript indicate a statistically significant difference (*p* < 0.05) between columns, between 2.5 g xanthan and 5 g xanthan addition. Capital letters (^A^, ^B^, ^C^, ^D^) in the superscript indicate a statistically significant difference (*p* < 0.05) between lines.

**Table 4:** The influence of water addition on biodegradable spoon texture.

Sample	Hardness (g)			
	50 mL Water	40 mL Water ^x^	30 mL Water	20 mL Water
4	542.75 ± 68.63 ^A^	655.16 ± 69.46	616.03 ± 88.03 ^A^	Not usable samples
4 ^S^	1172.04 ± 1259.93 ^B^	1553.75 ± 641.37	1002.1 ± 446.70 ^B^	Not usable samples

The letter ^S^ in the superscript indicates the dried samples. Lowercase letters (^x^) in the superscript indicate the sample according to the Table 1. Capital letters (^A^, ^B^) in the superscript indicate a statistically significant difference (*p* < 0.05) between lines.

**Table 5:** The influence of palm oil addition on the hardness of biodegradable spoons made of millet flour.

Sample	Hardness (g)	
	Without Oil	10 g of Oil
1P	710.27 ± 156.90	354.88 ± 37.75 ^AC^
2P	927.19 ± 122.72 ^aA^	616.56 ± 52.27 ^bBD^
3P	845.54 ± 119.60 ^A^	198.04 ± 92.00 ^AC^
1P ^S^	6204.51 ± 2711.98 ^a^	966.75 ± 391.60 ^bA^
2P ^S^	3672.03 ± 1392.37 ^a^	1196.97 ± 305.12 ^bB^
3P ^S^	3727.61 ± 1636.80	384.10 ± 128.95 ^CD^
4	542.75 ± 68.63 ^B^	396.03 ± 156.61 ^CD^
4 ^S^	1172.04 ± 1259.93	354.13 ± 209.09 ^CD^

The letter ^S^ in the superscript indicates the dried samples, the lowercase letter (*p*) in the superscript indicates the sample according to Table 1. Lowercase letters (^a^, ^b^) in the superscript indicate a statistically significant difference (*p* < 0.05) between columns. Capital letters (^A^, ^B^, ^C^, ^D^) in the superscript indicate a statistically significant difference (*p* < 0.05) between lines.

**Table 6:** Effect of palm oil addition on the hardness of biodegradable spoons containing xanthan.

Sample	Hardness (g)	
	Without Oil	5 g of Oil ^p^
2PX1 ^x^	1522.01 ± 320.51	1768.00 ± 646.27
2PX1 ^y^	1220.30 ± 505.66	1287.24 ± 424.49
2PX1 ^xS^	5951.24 ± 2395.60	2413.09 ± 403.82
2PX1 ^yS^	8003.48 ± 5160.20 ^a^	1232.29 ± 784.90 ^b^

The letter ^S^ in the superscript indicates dried samples. Lowercase letters (^x^, ^y^, ^p^) in the superscript indicate the sample according to Table 1. Lowercase letters (^a^, ^b^) in the superscript indicate a statistically significant difference (*p* < 0.05) between columns.

**Table 7:** The changes of texture parameters according to different concentrations of grape seed flour.

Sample	Hardness (g)		
	1:1 ^x^	3:1 ^y^	1:3 ^z^
1	849.22 ± 737.38	577.15 ± 106.09	344.9 ± 172.70

Lowercase letters (^x^, ^y^, ^z^) in the superscript indicate the sample according to Table 1.

**Table 8:** The texture of samples containing xanthan with different addition of grape seed flour.

Sample	Hardness (g)				
	0%	5% ^x^	10% ^y^		20% ^z^
9	2662.35 ± 601.62 ^ad^	2514.59 ± 248.92 ^acA^	1334.97 ± 231.09 ^bA^		1716.48 ± 121.96 ^bdA^
9 ^S^	-	9434.24 ± 2888.06 ^B^	9896.23 ± 902.34 ^B^		6346.92 ± 1764.98 ^B^
11	2763.61 ± 345.08	2924.31 ± 384.01 ^D^	2083.25 ± 363.38 ^A^		2742.46 ± 781.61 ^A^
11 ^S^	-	10,241.47 ± 523.23 ^aB^	6007.95 ± 804.17 ^bC^		6500.39 ± 1562.10 ^cB^
12	3186.86 ± 586.77 ^a^	1120.63 ± 68.71 bcC	1674.09 ± 319.29 ^bcA^		2214.9 ± 942.59 ^abcA^
12 ^S^	-	5496.79 ± 1735.10	4156.40 ± 1148.95 ^C^		3932.69 ± 1485.42

The letter ^S^ in the superscript indicates dried samples. Lowercase letters (^x^, ^y^, ^z^) in the superscript indicate the sample according to Table 1. Lowercase letters (^a^, ^b^, ^c^, ^d^) in the superscript indicate a statistically significant difference (*p* < 0.05) between columns. Capital letters (^A^, ^B^, ^C^, ^D^) in the superscript indicate a statistically significant difference (*p* < 0.05) between lines.

**Table 9:** Determination of total polyphenols and antioxidant activity by DPPH and FRAP.

Sample	Total Polyphenol Content (mg/g)	DPPH (%)	FRAP (µmol/g)
1P	0.007 ± 0.00 ^aA^	0.00 ^bAB^	1.51 ± 0.04 ^cA^
2PX1 ^y^	0.021 ± 0.00 ^aBD^	0.00 ^bAB^	0.73 ± 0.04 ^cB^
9 ^x^	0.012 ± 0.00 ^aC^	3.29 ± 0.17 ^bC^	5.22 ± 0.03 ^cC^
9 ^y^	0.021 ± 0.00 ^aBD^	10.49 ± 0.14 ^bDE^	10.92 ± 0.14 ^bD^
11 ^x^	0.015 ± 0.00 ^aE^	9.08 ± 0.10 ^bDE^	9.28 ± 0.11 ^bE^
11 ^z^	0.038 ± 0.00 ^aF^	25.91 ± 0.10 ^bF^	21.57 ± 0.14 ^bF^

Lowercase letters (^x^, ^y^, ^z^) in the superscript indicate the sample according to Table 1. Lowercase letters (^a^, ^b^, ^c^) in the superscript indicate a statistically significant difference (*p* < 0.05) between columns. Capital letters (^A^, ^B^, ^C^, ^D^, ^E^, ^F^) in the superscript indicate a statistically significant difference (*p* < 0.05) between lines.

## References

[1] Lebreton L., Andrady A. (2019). Future scenarios of global plastic waste generation and disposal. Palgrave Commun..

[2] Prata J.C., da Costa J.P., Lopes I., Duarte A.C., Rocha-Santos T. (2020). Environmental status of (micro) plastics contamination in Portugal. Ecotox. Environ. Safe..

[3] Hale R.C., Song B. (2020). Single-Use Plastics and COVID-19: Scientific Evidence and Environmental Regulations. Environ. Sci. Tech..

[4] Directive (EU) 2019/904 of the European Parliament and of the Council of 5 June 2019 on the Reduction of the Impact of Certain Plastic Products on the Environment. https://eur-lex.europa.eu/eli/dir/2019/904/oj.

[5] Patil H.N., Sinhal P. (2018). A study on edible cutlery: An alternative for conventional ones. Atithya J. Hosp..

[6] Adilah A.N., Jamilah B., Noranizan M.A., Hanani Z.N. (2018). Utilization of mango peel extracts on the biodegradable films for active packaging. Food Packag. Shelf Life.

[7] Behbahani B.A., Shahidi F., Yazdi F.T., Mortazavi S.A., Mohebbi M. (2017). Use of Plantago major seed mucilage as a novel edible coating incorporated with Anethum graveolens essential oil on shelf life extension of beef in refrigerated storage. Int. J. Biol. Macromol..

[8] Tomadoni B., Cassani L., Ponce A., Moreira M.D.R., Agüero M.V. (2016). Optimization of ultrasound, vanillin and pomegranate extract treatment for shelf-stable unpasteurized strawberry juice. LWT-Food Sci. Tech..

[9] Mohammadi M., Sadeghnia N., Azizi M.H., Neyestani T.R., Mortazavian A.M. (2014). Development of gluten-free flat bread using hydrocolloids: Xanthan and CMC. J. Ind. Eng. Chem..

[10] Prajapati V.D., Jani G.K., Moradiya N.G., Randeria N.P. (2013). Pharmaceutical applications of various natural gums, mucilages and their modified forms. Carbohyd. Polym..

[11] Encina-Zelada C.R., Cadavez V., Monteiro F., Teixeira J.A., Gonzales-Barron U. (2018). Combined effect of xanthan gum and water content on physicochemical and textural properties of gluten-free batter and bread. Food Res. Int..

[12] Jan R., Saxena D.C., Singh S. (2016). Physico-chemical, textural, sensory and antioxidant characteristics of gluten–Free cookies made from raw and germinated Chenopodium (Chenopodium album) flour. LWT-Food Sci. Tech..

[13] Moses J.A., Norton T., Alagusundaram K., Tiwari B.K. (2014). Novel drying techniques for the food industry. Food Eng. Rev..

[14] Arepally D., Reddy R.S., Goswami T.K., Datta A.K. (2020). Biscuit baking: A review. LWT.

[15] De La Hera E., Rossel C.M., Gomez M. (2014). Effect of water content and flour particle size on gluten-free bread quality and digestibility. Food. Chem..

[16] Manohar R.S., Rao P.H. (1997). Effect of mixing period and additives on the rheological characteristics of dough and quality of biscuits. J. Cereal Sci..

[17] Detry R., Van Hoed V., Sterckx J., Deledicque C., Sato K., Blecker C., Danthine S. (2021). Physicochemical Properties of Palm Oil-Based Puff Pastry Model Margarines Related to Their Baking Performance in Long-Term Storage. Eur. J. Lipid Sci. Tech..

[18] Sirohi R., Tarafdar A., Singh S., Negi T., Gaur V.K., Gnansounou E., Bhartiraja B. (2020). Green processing and biotechnological potential of grape pomace: Current trends and opportunities for sustainable biorefinery. Bioresour. Tech..

[19] Ross C.F., Hoye C., Fernandez-Plotka V.C. (2011). Influence of heating on the polyphenolic content and antioxidant activity of grape seed flour. J. Food Sci..

[20] Antonic B., Dordevic D., Jancikova S., Holeckova D., Tremlova B., Kulawik P. (2021). Effect of Grape Seed Flour on the Antioxidant Profile, Textural and Sensory Properties of Waffles. Processes.

[21] Kalinová J. (2007). Nutritionally important components of proso millet (Panicum miliaceum L.). Food.

[22] Levent H., Sayaslan A., Yeşil S. (2021). Physicochemical and sensory quality of gluten-free cakes supplemented with grape seed, pomegranate seed, poppy seed, flaxseed, and turmeric. J. Food Processs. Preserv..

[23] Nakov G., Brandolini A., Hidalgo A., Ivanova N., Stamatovska V., Dimov I. (2020). Effect of grape pomace powder addition on chemical, nutritional and technological properties of cakes. LWT.

